# Evaluation of the biocompability and corrosion activity of resorbable CaMgZnYbBAu alloys

**DOI:** 10.1038/s41598-022-25069-6

**Published:** 2022-12-05

**Authors:** Dawid Szyba, Robert Kubina, Katarzyna Młynarek-Żak, Adrian Radoń, Aneta Kania, Rafał Babilas

**Affiliations:** 1 Department of Engineering Materials and Biomaterials, Faculty of Mechanical Engineering, Silesian University of Technology, Konarskiego 18a, 44-100 Gliwice, Poland; 2 Department of Pathology, Faculty of Pharmaceutical Sciences in Sosnowiec, Medical University of Silesia in Katowice, Ostrogórska 30, 41-200 Sosnowiec, Poland; 3 Silesia LabMed: Centre for Research and Implementation, Medical University of Silesia in Katowice, 18 Medyków Str, 40-752 Katowice, Poland

**Keywords:** Biomaterials, Metals and alloys

## Abstract

Calcium-based alloys can be promising candidates for use as biodegradable implants because of attractive properties as mechanical, corrosive, and biocompatible. In the work, the biocompatibility authors discussed the results of the Ca_32_Mg_12_Zn_38_Yb_18−x_B_x_ (x = 0, 1, 2, 3 at.%) and Ca_32_Mg_12_Zn_38_Yb_18−2x_B_x_Au_x_ (x = 1, 2 at.%) alloys. The tests were performed using a MTT assay. The corrosion behavior of such Ca-based alloys in PWE fluid at 37 °C was studied and compared with the results in Ringer’s solution from previous works. Electrochemical tests were presented by open circuit potential and potentiodynamic curves. Different concentrations of boron and gold in the alloys caused changes in the corrosion results. The best corrosion resistance in PWE solution was observed for the Ca-based alloy with 2 at.% Au due to the lowest value of the corrosion current density (*j*_corr_), equal to 10.6 µA·cm^−2^. A slightly higher value of *j*_corr_ was obtained for the Ca_32_Mg_12_Zn_38_Yb_15_B_3_ alloy with the lowest roughness values. The results of the cytotoxicity tests also showed that the alloy with 3 at.% boron was characterized by the highest cell viability. The investigation results discussed in the work allow us to suggest that the presented calcium alloys with 3 at.% of B, and 2 at.% of Au addition may be promising materials for the use in implantology.

## Introduction

Modern methods of treating diseases of the musculoskeletal system place high demands on materials used in medicine, including those used for bone implants fabrication. These materials should have many properties (e.g. mechanical, physicochemical, corrosive), but most of all they should be biocompatible (biotolerant) and non-toxic^1^.

In recent years, biocompatible and biodegradable implants, such as polymer, ceramic, and metallic (e.g. magnesium-based alloys), which degrade slowly after implantation, have been extensively researched. This kind of biomaterial is more beneficial because it reduces the cost of healthcare and eliminates the next surgery to remove implants^2–4^. Among these biomaterials proposed for bone implants, calcium-based alloys are found. In the literature, there is not as much work on the use of Ca-based alloys with various additives, such as magnesium, zinc, ytterbium, boron, and gold in implantology. Therefore, it seems to be very interesting to present the behavior of such alloys in the human body environment, especially from a biological point of view.

It should be mentioned that calcium and most calcium-based alloying elements are present in the human body as micronutrients (e.g. zinc, boron) and macronutrients (e.g. calcium, magnesium). These are elements necessary for healthy functioning (also known as bioelements). Calcium is one of the minerals present in the body, necessary for its proper functioning. The total amount of calcium in an adult human body is approximately 1200 g, which is 1.5% of body weight. 99% of Ca is present in bones in the form of bound to apatite, the remainder is present in the ionized form in the intracellular and extracellular fluid. Furthermore, calcium is an inexpensive element with low density (1.55 g/cm^3^), which is essential for the application of short-term calcium-based alloys in orthopedic implants^5^. Biodegradable magnesium is highly biocompatible. It has mechanical properties similar to natural bones. Furthermore, Mg as an alloying element improves mechanical properties and corrosion resistance. This extremely important macroelement has a multidirectional effect on the human body^5^. Therefore, it has been used in the treatment and prevention of many diseases. Most of all, magnesium is essential for the proper structure of bone tissue. In addition to calcium, vitamin D, and phosphorus, it affects proper bone mineralization, protecting human against osteoporosis. Mg is responsible for the activity of osteoblasts and osteoclasts, i.e. cells that are involved in bone metabolism^4^.

The next element, zinc, is a catalyst for many reactions. It participates in the transformation of proteins, fats, and carbohydrates. Zn is necessary to maintain the stability of cell membranes. It has regulatory and structural functions^6^. In addition, zinc participates in energy transformations and is an element necessary to maintain proper body weight. In recent years, ytterbium, as a rare earth element, has been used as a promising alloy additive to modify microstructures and improve the mechanical properties of traditional magnesium alloys^7^. It has also been noted that ytterbium salts stimulate metabolism. Yu et al.^8^ stated that the addition of 4% Yb to the Mg_66_Zn_30_Ca_4_ alloy decreases the cytotoxicity in in vitro studies. Boron is a very important element that has a positive effect on immunity and has anti-inflammatory properties. It has a positive effect on bone health and prevention of arthritis, playing a potential role in bone formation^9,10^, calcium, vitamin D, magnesium, phosphorus, and fluoride metabolism (ensuring that these micronutrients are at the correct levels and that vitamins are better absorbed). These make boron useful in the prevention of osteoporosis and rheumatic diseases. Some works suggest that boron may be a natural anticancer agent^11^. Gold as an alloying additive also has anticancer properties^12^. The results show that Au compounds are more cytotoxic to cancer cells than to healthy ones^13^. In addition, gold compounds show healing properties. Due to reactions with skin lipids, gold nanoparticles are able to open the stratum corneum and penetrate through it^14^, and the degree of penetration depends on their physicochemical properties^15^.

The correct selection of the composition of a potential biomaterial is extremely important. Improper selection of a metal material or alloy used in an implantology can result in an allergic reaction in the body, known as metallosis^16^. It is caused by the passage of implant fragments into its environment (which is usually caused by abrasive wear on the implant surface), migration of corrosion products from the implant surface to the surrounding environment, or by the reaction of metal ions in contact with human body fluids. Metallosis can occur in several ways. There is quiet, sharp, and discreet metallosis.

The basic property of the material, which determines its suitability for biomedical applications, is biotolerance. Biocompatibility evaluation^17^, according to an international standard, requires in vitro tests on isolated cells or tissues, as well as in vivo tests in animals and preclinical trials^18^. In vitro tests are a way to initially determine the behavior of living cells in the presence of the tested biomaterial. One of the basic in vitro tests is a biological cytotoxicity of materials used in medicine. Toxic substances released by the material in contact with tissues or body fluids can damage the cell membrane, change the metabolic activity of cells, and damage the genetic material of the cell. Cytotoxicity is the evaluation of the effect of a potential biomaterial on cells observed under a microscope after a predetermined period of exposure or by the activity of enzymes proving the viability of the cells. However, it should be mentioned that the cytotoxicity test does not ensure the maintenance of all the conditions of the physiological environment, but it is a study evaluating the behavior of living cells in contact with the biomaterial. Each medical device or the materials used for its production, also intended for implantation, are compliant with biological tissues, cells, and body fluids. The types of methods for in vitro and in vivo tests related to the assessment of biological compliance of medical devices constitute the content of the applicable PN-EN ISO 10993-1:2010 standard (Biological assessment of medical devices—Part 1: Evaluation and testing in the risk management process). The selection of method depends on the use of the product, the time the implant spent in the human body, and the type of contact (including external, internal contact, intact skin, mucous membranes, contact with blood, bone tissue, tissue fluids, etc.). Taking into consideration the time of contact of the biomaterial with the body, this standard classifies medical devices into those that remain in the patient's body for no longer than 24 h, over 24 h, but no longer than 30 days, and which are in constant contact, i.e. over 30 days. Among the biocompatibility tests, depending on the duration of contact with the organism and the intended use of the biomaterial, the following can be distinguished^19^:

 − in vitro cytotoxicity,
 − sensitizing effect,
 − irritating effect or intracutaneous reactivity,
 − acute systemic toxicity,
 − subacute and subchronic toxicity,
 − genotoxicity,
 − post-implantation reaction and compatibility with blood.

Different compounds interact with cells in different ways, disrupting or not their life processes. One of the basic documents for the assessment of the toxicity of medical devices is the PN-EN ISO 10993-5:2009 standard. Biological evaluation of medical devices—Part 5: In vitro cytotoxicity tests. Cytotoxic activity can be determined on the basis of changes that occur in cells under the influence of the studied material in relation to the control sample^20^. The control sample is a cell culture carried out without biomaterial or with material in the generally known cytotoxic concentration range. When determining the viability of cells on the basis of the number of dead cells in contact with the biomaterial, this study may disqualify the biomaterial for further use. One of the most widely used assays to test cell viability is the MTT assay^19^. This test does not provide information on the type of toxins that occur. It determines the biological effects known as the cytotoxicity of materials of different composition^21^.

In this context, the aim of the work was to study the biological and corrosion behavior of six Ca–Mg–Zn–Yb alloys with various additions of boron and gold. In vitro cytotoxicity tests do not allow one to recreate all the conditions present in a living organism, but they are an excellent way to initially determine the behavior of living cells in the contact with tested potential biomaterial. Moreover, the authors wanted to verify which of the proposed alloy compositions is more cytocompatible and corrosion resistant.

## Materials and methods

### Materials characterisation

The investigations were carried out on Ca_32_Mg_12_Zn_38_Yb_18-x_B_x_ alloys (x = 0, 1, 2, 3 at.%) and Ca_32_Mg_12_Zn_38_Yb_18-2x_B_x_Au_x_ alloys (x = 1, 2 at.%). Base alloys were produced by induction melting of elements with a purity of 99.9%. The alloys were remelted several times to obtain a homogeneous composition of ingots. All alloys were cast under an inert atmosphere. The casting chamber was purged with argon, but this did not provide enough air reduction in the chamber. Rapidly cooled samples in the form of plates with a length and width of 10 mm and a thickness of 1 mm were cast using copper mold casting.

The topography and roughness measurements of the samples were performed using the ZEISS LSM Exciter 5 confocal microscope (Zeiss, Oberkochen, Germany) with an observation system of 4 lasers of wavelength in the range of 405 to 633 nm. The device was equipped with a ZEN image acquisition and analysis system. The surfaces of the samples were mechanically polished with SiC papers from 500 to 2400 gradation before the observations by confocal microscope. Then, they were polished with a diamond suspension and cleaned with alcohol.

### Electrochemical measurements

The corrosion resistance was assessed on the basis of electrochemical tests. Measurements were carried out in PWE (NaCl—5.75 g·cm^−3^, KCl—0.38 g·cm^−3^, CaCl_2_·6H_2_O—0.394 g·cm^−3^, MgCl_2_·6H_2_O—0.2 g·cm^−3^, CH_3_COONa—4.62 g·cm^−3^, Na_3_C_6_H_5_O_7_·2H_2_O—0.9 g·cm^−3^) and Ringer’s solution (8.6 g/dm^3^ NaCl, 0.3 g/dm^3^ KCl, and 0.48 g/dm^3^ CaCl_2_–6H_2_O) at 37 °C using an Autolab 302 N potentiostat (Metrohm AG, Herisau, Switzerland). The conditions of the experiment were similar to the natural environment inside the organism. The potentiostat was equipped with a cell containing the reference electrode (saturated calomel electrode) and the counter electrode (platinum rod). The CaMgZnYbBAu alloys in the form of plates were tested with 3600 s of open circuit potential (*E*_OCP_). Potentiodynamic curves with Tafel’s extrapolation were recorded in a potential range from *E*_OCP_ − 250 mV to *E*_OCP_ + 250 mV, a scan rate was 1 mV· s^−1^. The corrosion potential (*E*_corr_) and corrosion current density (*j*_corr_) were determined by Tafel’s extrapolation using cathodic and anodic branches of the polarization curves.

### Corrosion products analysis

After 48 h of immersion in Ringer’s and PWE solutions, the surfaces of the corroded samples were observed with corrosion products using scanning electron microscope (SEM, Thornwood, New York, USA), which was equipped with an energy dispersive X-ray spectrometer (EDS) detector. Fourier transform infrared (FTIR) spectroscopy was also used to analyze the corrosion products. FTIR spectra were recorded at room temperature for the Ca_32_Mg_12_Zn_38_Yb_15_B_3_ and Ca_32_Mg_12_Zn_38_Yb_14_B_2_Au_2_ alloys using a Nicolet 6700/8700 FTIR spectrometer (Thermo Fisher Scientific, Waltham, USA). Before the measurements, the corrosion products were collected from the surface of the immersed samples and mixed together with dry KBr. The samples were performed in transmission mode in a mid infrared range of 4000–400 cm^−1^.

### Cytotoxicity tests

Human osteosarcoma cells (U2-OS) derived from the ATCC+ HTB-96™ collection were used to evaluate the cytocompatibility of the experimental alloys. Cells were cultured using McCoy’s 5a Medium Modified medium, supplemented with 10% Fetal Bovine Serum (FBS), and 100 U ml^−1^ penicillin and 100 µg·ml^−1^ streptomycin. The cells were stored in an atmosphere of 37 °C saturated with water vapor and enriched with 5% CO_2_. The medium was changed every 2–3 days and cells were passed at 90% confluence. Biocompatibility was performed by indirect contact. Samples in the form of plates for cytotoxicity assessment were 1 mm thick. They were sterilized in an autoclave at 121 °C for 21 min at a steam pressure of 1.5 bar.

According to ISO 10993-5:1999, the extraction medium was prepared using serum-free culture medium with a surface area to extraction medium ratio of 1 cm^2^/ml in an atmosphere saturated with steam with water supplemented with 5% CO_2_ at 37 °C for 24 and 72 h. The extract was stored at 4 °C prior to the cytotoxicity test. Pure extract (100%) diluted with culture medium 1:1, 1:2, 1:4, and 1:8 were used for the tests.

**Figure 1:**
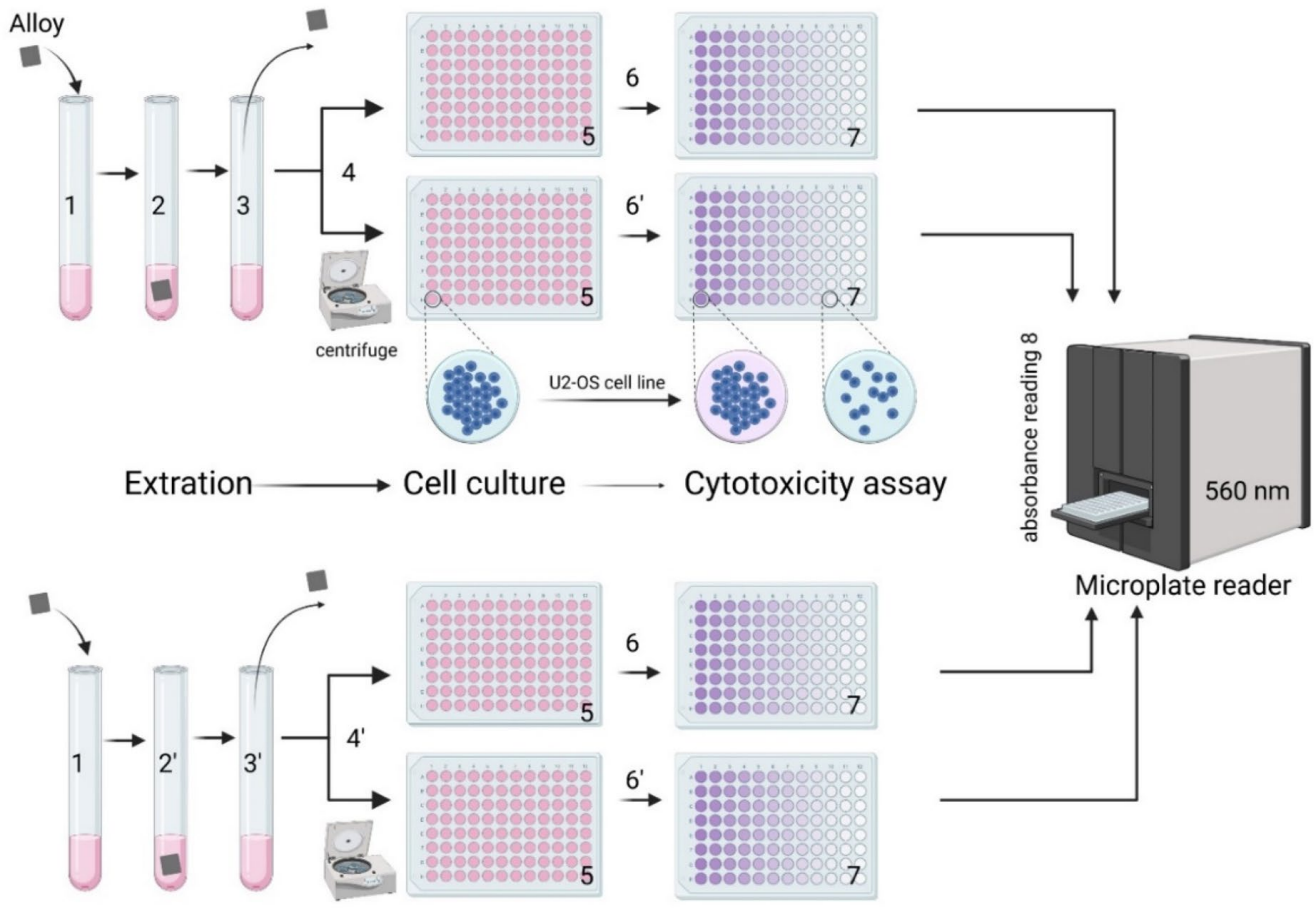
Schematic ilustration of cytotoxicity test according with ISO 10993-5:1999.

Cells were incubated in 96-well culture plates at a density of 5 × 10^3^ cells/100 µl medium in each well and incubated for 48 h to allow cell adhesion and log growth to be achieved. After this time, the medium was replaced with 100 μl of extraction medium and incubated for 24 and 48 h. After this time, the supernatant was decanted, and 100 µl of MTT reagent dissolved in medium at a final concentration of 1 mg/ml was added to each well. The samples were incubated with the MTT reagent for 4 h at 37 °C. The medium was then gently decanted and 200 µl of DMSO was added to dissolve the resulting formazan crystals. The spectrophotometric absorbance of the samples was measured with a microplate reader (BioTek ELx800) at 570 nm with a reference wavelength of 630 nm. The schematic illustration of the cytotoxicity test provided in this work is presented in Fig. 1. (1, 2) The extraction medium was prepared using a ratio of alloy culture medium with a surface area to extraction medium ratio of 1 cm^2^/ml for 24 h (2) and 72 h (2’). (3) After the alloy extraction period the sample was removed and the medium was centrifuged (4). (5) In O2-OS cells the indirect extract was added and the cells were incubated for 24 h or 48 h. (6) After the incubation period 24 h (6) or 48 h (6’) the supernatant was decanted and 100 µl of MTT reagent dissolved in medium was added to each well. Microplate was incubated for 4 h in a humidified atmosphere, such as + 37 °C, 5% CO_2_. The medium was then gently decanted and 200 µl of DMSO was added to dissolve the crystals. (7) A 96 well plate after the MTT assay. Metabolism of MTT to a formazan salt by viable cells, as shown in a 96-well plate. The decrease in the number of viable and metabolically active cells results in the decreasing intensity of the purple color observed.

## Results and discussion

**Figure 2:**
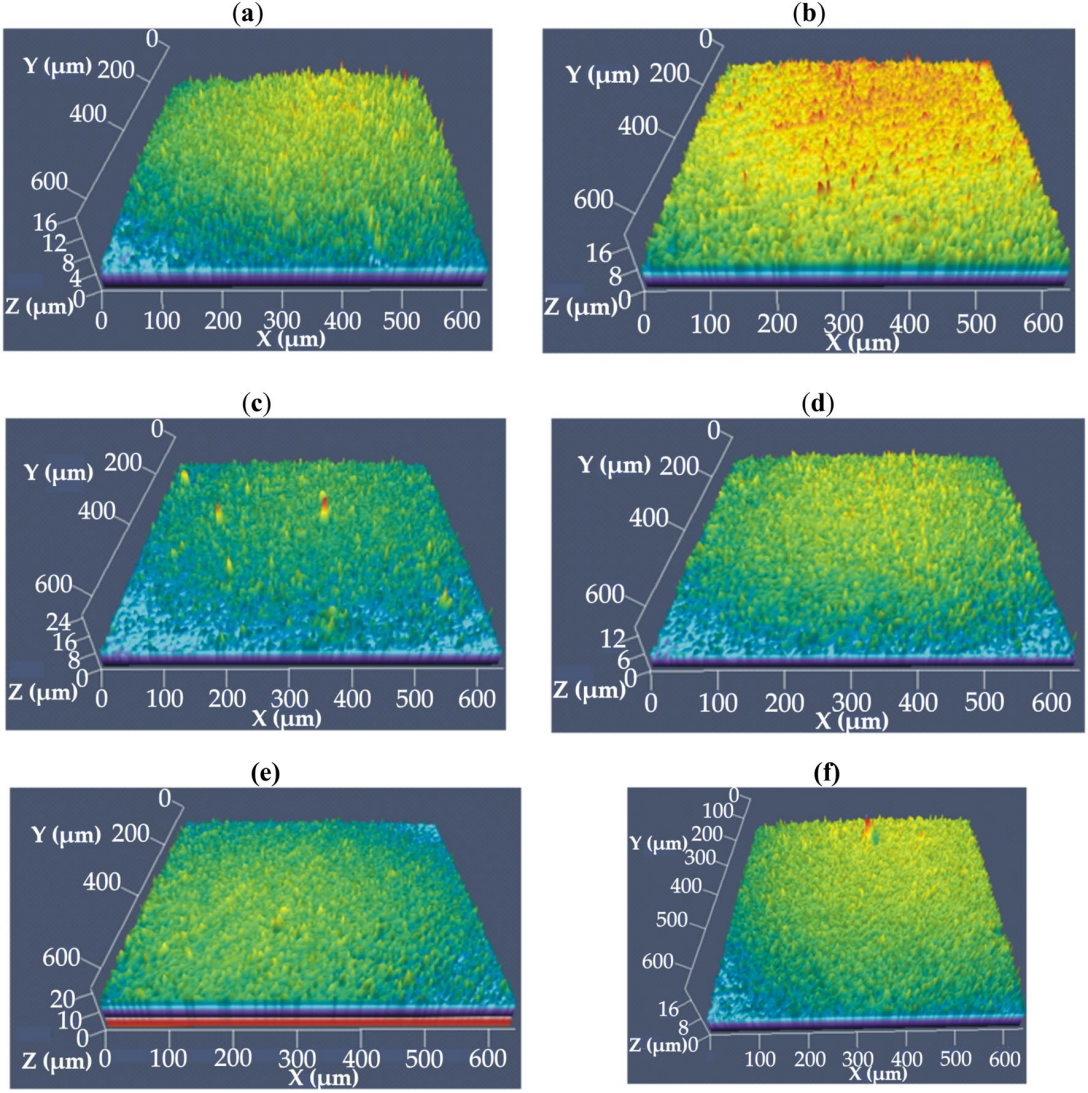
Confocal images of the Ca_32_Mg_12_Zn_38_Yb_18-x_B_x_ (x = 0, 1, 2, 3 at.%) and Ca_32_Mg_12_Zn_38_Yb_18-2x_B_x_Au_x_ (x = 1, 2 at.%) alloys in a form of plate: (**a**) Ca_32_Mg_12_Zn_38_Yb_18;_; (**b**) Ca_32_Mg_12_Zn_38_Yb_17_B_1;_; (**c**) Ca_32_Mg_12_Zn_38_Yb_16_B_2_; (**d**) Ca_32_Mg_12_Zn_38_Yb_15_B_3_; (**e**) Ca_32_Mg_12_Zn_38_Yb_16_B_1_Au; (**f**) Ca_32_Mg_12_Zn_38_Yb_14_B_2_Au_2_.

The resorbable CaMgZnYbBAu alloys in a form of plates had an amorphous structure (Ca_32_Mg_12_Zn_38_Yb_18,_ Ca_32_Mg_12_Zn_38_Yb_16_B_2_), an amorphous structure with some crystalline phases, such as CaZn, MgZn (Ca_32_Mg_12_Zn_38_Yb_17_B_1_, Ca_32_Mg_12_Zn_38_Yb_15_B_3_) and an amorphous structure with CaZn, MgZn and CaZn_2_ crystalline phases (Ca_32_Mg_12_Zn_38_Yb_16_B_1_Au, Ca_32_Mg_12_Zn_38_Yb_14_B_2_Au_2_). All identified phases were embedded in the amorphous structure^22–24^. The structure studies of the alloys were completed by the surface topography analysis. The surface topography of the alloys studied was observed using a confocal microscope (Fig. 2). Measurements were carried out in an area of 3.6 × 10^5^ µm^2^ (600 × 600 µm). The surfaces of the Ca_32_Mg_12_Zn_38_Yb_18-x_B_x_ alloys (x = 0, 1, 2, 3 at.%) and Ca_32_Mg_12_Zn_3__8_Yb_18-2x_B_x_Au_x_ alloys (x = 1, 2 at.%) were similar in their topography, and had a granular structure. This was in agreement with small changes in the structure of these alloys. It is well known that the surface roughness values of alloys influence the corrosion resistance and cell viability. Any scratches on the metal surface may increase stress, i.e., reduce crack resistance. This leads to stress corrosion cracking or hydrogen embrittlement, which finally causes premature failure of biodegradable implants during operation^25,26^. Many works, especially on Mg-based alloys, show that initial surface roughness influences the intensity of corrosion behavior^27,28^. Additionally, for orthopedic implants that come into contact with blood, it is important to minimize surface roughness.

In addition, a surface roughness analysis was also performed. The surface roughness parameters (roughness average—*R*_a_ and root mean square—*R*_S_) of the Ca-based alloys were determined and listed in Table 1. The analysis showed that the roughness values for all of the alloys studied were similar. However, the lowest roughness values were obtained for the Ca_32_Mg_12_Zn_38_Yb_15_B_3_ and Ca_32_Mg_12_Zn_38_Yb_18_ alloys. These may suggest that these alloys were more corrosion resistant, because the lower the roughness values, the higher the corrosion resistance^29,30^. It should also be noted that corrosion resistance is dependent not only on the surface roughness but also on the ability of the alloy to form a protective passive film on its surface^30^. Mitchell et al.^27^ studied the effect of surface roughness on the corrosion behavior of AZ31 Mg alloys for 672 h of immersion in a solution of 3.5 wt.% NaCl. The results have shown that the corrosion rate and the formation of the oxide layer (MgO) on the surface of the AZ31 alloy were proportional to the average roughness value (*R*_a_) of the samples. The surface of the roughened sample had pores and microcracks that were potential places for the corrosion initiation. Moreover, in this sample, the dissolution of the metals was greater, resulting in mass reduction. In the studies performed by Walter et al.^28^ the passivation behavior of the AZ91 alloy with different surface roughness was presented. The authors suggested that the surface roughness of the Mg alloy played a critical role in the corrosion resistance in the 3.5% NaCl environment. They confirmed that the sample with a smoother surface has shown a greater tendency to passivation and pitting resistance than the sample with a higher surface roughness.

**Table 1:** Surface roughness parameters for the Ca_32_Mg_12_Zn_38_Yb_18-x_B_x_ (x = 0, 1, 2, 3 at.%) and Ca_32_Mg_12_Zn_38_Yb_18-2x_B_x_Au_x_ (x = 1, 2 at.%) alloys.

Sample	Designation	Roughness average, *R*_a_, µm	Root mean square, *R*_S_, µm
Ca_32_Mg_12_Zn_38_Yb_18_	B0	1.11 (± 0.025)	1.42 (± 0.023)
Ca_32_Mg_12_Zn_38_Yb_17_B_1_	B1	1.44 (± 0.021)	1.84 (± 0.022)
Ca_32_Mg_12_Zn_38_Yb_16_B_2_	B2	1.37 (± 0.021)	1.79 (± 0.02)
Ca_32_Mg_12_Zn_38_Yb_15_B_3_	B3	1.09 (± 0.021)	1.39 (± 0.023)
Ca_32_Mg_12_Zn_38_Yb_16_B_1_Au	Au1	1.26 (± 0.03)	1.62 (± 0.028)
Ca_32_Mg_12_Zn_38_Yb_14_B_2_Au_2_	Au2	1.4 (± 0.016)	1.76 (± 0.019)

**Figure 3:**
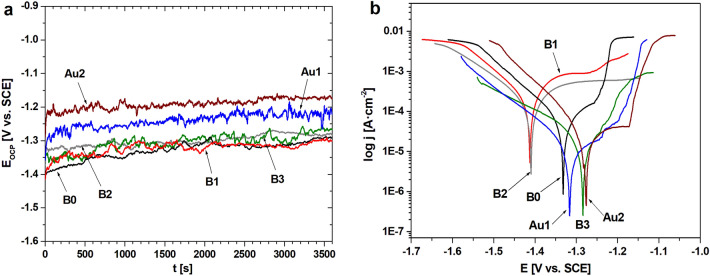
Changes of open-circuit potential with time (**a**) and polarization curves (**b**) of CaMgZnYbBAu alloys in a form of plates in PWE solution at 37 °C.

Electrochemical tests in PWE solution were presented in both the open circuit potential (*E*_OCP_) and the potentiodynamic curves (Fig. 3). Changes in the *E*_OCP_ potential were measured to evaluate protective properties of the alloys Ca_32_Mg_12_Zn_38_Yb_18−x_B_x_ (x = 0, 1, 2, 3 at.%) and Ca_32_Mg_12_Zn_38_Yb_18−2x_B_x_Au_x_ (x = 1, 2 at.%). The curves determined for a stationary potential as a function of immersion time indicate that CaMgZnYbBAu alloys in plate form were active in PWE solution (Fig. 3a). Furthermore, the alloys studied were characterized by significant fluctuations. It can also be observed that the Ca_32_Mg_12_Zn_38_Yb_14_B_2_Au_2_ and Ca_32_Mg_12_Zn_38_Yb_16_B_2_ alloys were more stable in PWE solution compared to the others. The *E*_OCP_ potentials were similar for all CaMgZnYbBAu alloys. They were in the range of − 1.3 V to − 1.2 V. Furthermore, the differences in *E*_OCP_ potential between alloys with 1 and 2 at.% Au were not greater than 50 mV. The same situation was observed for the alloys with and without boron content. Polarization curves for alloys with 1 and 2 at.% of B were located at higher current values above 100 μA/cm^2^ (Fig. 3b). This may suggest that the Ca_32_Mg_12_Zn_38_Yb_17_B_1_ and Ca_32_Mg_12_Zn_38_Yb_16_B_2_ alloys exhibit lower corrosion resistance. The extrapolation of the polarization curves using the Tafel’s method allowed us to determine the corrosion current density (*j*_corr_) and polarization resistance (*R*_p_) of the CaMgZnYbBAu alloys (Table 2).

**Table 2:** Comparison of electrochemical properties for Ca_32_Mg_12_Zn_38_Yb_18-x_B_x_ (x = 0, 1, 2, 3 at.%) and Ca_32_Mg_12_Zn_38_Yb_18-2x_B_x_Au_x_ (x = 1, 2 at.%) alloys in Ringer’s solution and PWE fluid at 37 °C.

Alloy	Solution	Open circuit potential (*E*_OCP_) [mV]	Corrosion potential (*E*_corr_) [mV]	Corrosion current density (*j*_corr_) [μA/cm^2^]	Polarisation resistance (*R*_p_) [kΩcm^2^]	Ref
Ca_32_Mg_12_Zn_38_Yb_18_	PWE fluid	− 1289	− 1331	96.7	0.37	This work
	Ringer’s solution	− 1305	− 1345	190.0	0.32	^23^
Ca_32_Mg_12_Zn_38_Yb_17_B_1_	PWE fluid	− 1302	− 1413	443.4	0.09	This work
	Ringer’s solution	− 1312	− 1410	356.2	0.05	^22^
Ca_32_Mg_12_Zn_38_Yb_16_B_2_	PWE fluid	− 1284	− 1395	175.7	0.18	This work
	Ringer’s solution	− 1308	− 1313	128.9	0.63	^22^
Ca_32_Mg_12_Zn_38_Yb_15_B_3_	PWE fluid	− 1267	− 1286	29.0	1.32	This work
	Ringer’s solution	− 1258	− 1335	174.7	0.49	^22^
Ca_32_Mg_12_Zn_38_Yb_16_B_1_Au_1_	PWE fluid	− 1223	− 1319	18.0	1.92	This work
	Ringer’s solution	− 1238	− 1305	18.45	0.67	^24^
Ca_32_Mg_12_Zn_38_Yb_14_B_2_Au_2_	PWE fluid	− 1176	− 1277	10.6	1.01	This work
	Ringer’s solution	− 1221	− 1260	8.79	1.82	^24^

The lowest value of corrosion current density was observed in PWE fluid, equal to 10.6 µA·cm^−2^, was observed for the Ca-based alloy with 2 at.% Au. The slightly higher values of *j*_corr_ (equal to 18 and 29 µA·cm^−2^) were obtained for alloys with 1 at.% Au and 3 at.% B. These alloys (Ca_32_Mg_12_Zn_38_Yb_15_B_3_, Ca_32_Mg_12_Zn_38_Yb_16_B_1_Au_1_, Ca_32_Mg_12_Zn_38_Yb_14_B_2_Au_2_) were characterized by higher polarization resistance that indicate better corrosion resistance compared to alloys B0, B1 and B2. The low *j*_corr_ and high *R*_p_ of the Ca_32_Mg_12_Zn_38_Yb_15_B_3_ alloy is consistent with the small roughness values.

**Figure 4:**
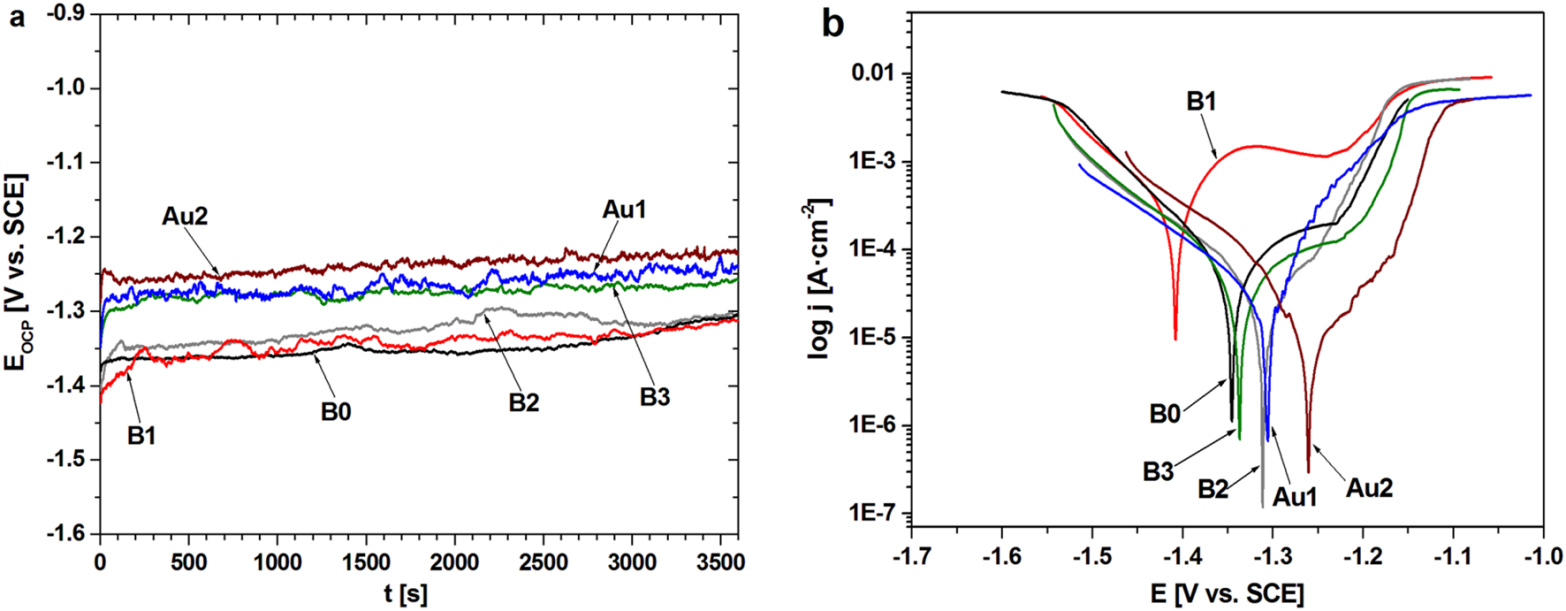
Changes of open-circuit potential with time (**a**) and polarization curves (**b**) of CaMgZnYbBAu alloys in a form of plates in Ringer’s solution at 37 °C^22–24^.

For comparison, the changes in *E*_OCP_ with time and polarization curves for all alloys in Ringer’s solution were presented in Fig. 4. The lowest values of corrosion current density in Ringer’s solution were observed for alloys with 1 and 2 at.% addition of Au^22–24^.

The surface roughness of implants is assumed to be an important factor in the osseointegration of these materials^31^. This parameter is responsible for the degradation of metallic materials^32,33^. Studies on the impact of surface roughness on the corrosive behavior of magnesium alloy (AZ91) in a chloride-containing environment at room temperature showed that the pitting corrosion of the alloy critical depends on the roughness of the surface^28^.

The smoother surface of Ca alloy with 3 at.% of B improved the corrosion resistance in the chloride environment. Therefore, it is very important to design alloys with optimal surface roughness to improve their corrosion properties^34,35^. Reddy et al.^34^ studied the effect of surface roughness on the corrosion behavior in 3.5 wt.% NaCl of two AZ31 and AZ80 alloys. The researchers stated that the higher corrosion resistance of AZ80 compared to AZ31 was due to lower surface roughness. Hence, the lower surface roughness of the samples improves surface integrity in such a way as to increase corrosion resistance^36^. Similar results were obtained in terms of the correlation between surface roughness and corrosion resistance of biomedical materials for stainless steel and titanium alloys^37–39^.

The corrosion resistance of orthopedic implants depends on the alloy additives. Calcium as a base metal increases strength and hardness. The influence of Ca on the microstructure of the alloy and electrochemical behavior can also be seen^4^. This element is important in the context of the kinetics of implant degradation because it increases their resorption. Magnesium in Ca-based alloys has mechanical properties similar to those of human bones, but it is characterized by a high corrosion rate^4^. This disadvantage of Mg can be reduced by the addition of Zn, Yb, B, and Au metals to Ca-based alloys. The addition of Zn causes grain refinement and the formation of secondary phases, which affects the mechanical and corrosive properties of Mg alloys, because zinc improves the corrosion potential of the biomedical material^40^. Moreover, Zn transforms impurities such as iron, copper, and nickel into harmless intermetallic compounds, thus reducing their corrosion-promoting effects^7^. The physical and chemical properties of Yb as a rare-earth element are similar to those of calcium. The authors^40^ studied the corrosion behavior in simulated body fluid (SBF) of amorphous Mg–Zn–Yb–Ag alloys with different contents of ytterbium. They stated that the addition of 4 at.% of Yb significantly improved the corrosion resistance compared to the other alloys tested. In the work^24^ there was observed that ytterbium has a positive effect on the corrosion resistance of Ca–Mg–Zn alloys in chloride-rich Ringer solution. In addition, the authors in^24^ suggested that the addition of boron and gold to a Ca-based alloys slowed the corrosion rate (due to creating a barrier among Ca, B, and Cl ions and forming a thick layer of corrosion products) and decreased the evolution of H_2_.

Furthermore, Hernandez-Rodriguez et al.^41^ tested the effect of different boron content (0.06, 0.25, 0.5 and 1 wt.%) in a CoCrMo biocompatible alloy on corrosion resistance in phosphate buffered saline (PBS). They observed an improvement in corrosion resistance with increased B content. Similar electrochemical corrosion results were achieved during immersion in Ringer’s solution^22–24^. The good anticorrosive properties of such alloys were also resulted from the addition of gold as an alloying element^42^ or as nanoparticles located in an applied coating^43,44^. Lee et al.^42^ studied the corrosion resistance of Ti-xAu alloys (x = 5, 10, 15, 20 and 40 wt.%) in 0.9% NaCl solution at 37 °C. They stated that the Ti-20Au alloy exhibited the lowest E_corr_ value, equal to − 278.33 mV, but the lowest corrosion current density was observed for the Ti-5Au alloy. Thin gold films (with a thickness of about 50 and 100 nm) which have been applied to Ni–Cr–Mo alloys using magnetron sputtering technique were tested by Wadullah et al.^44^. The corrosion behavior of the samples was studied in an artificial saliva solution at 37 °C. The authors observed lower values for corrosion current densities and higher values for corrosion potentials for alloys coated with gold.

**Figure 5:**
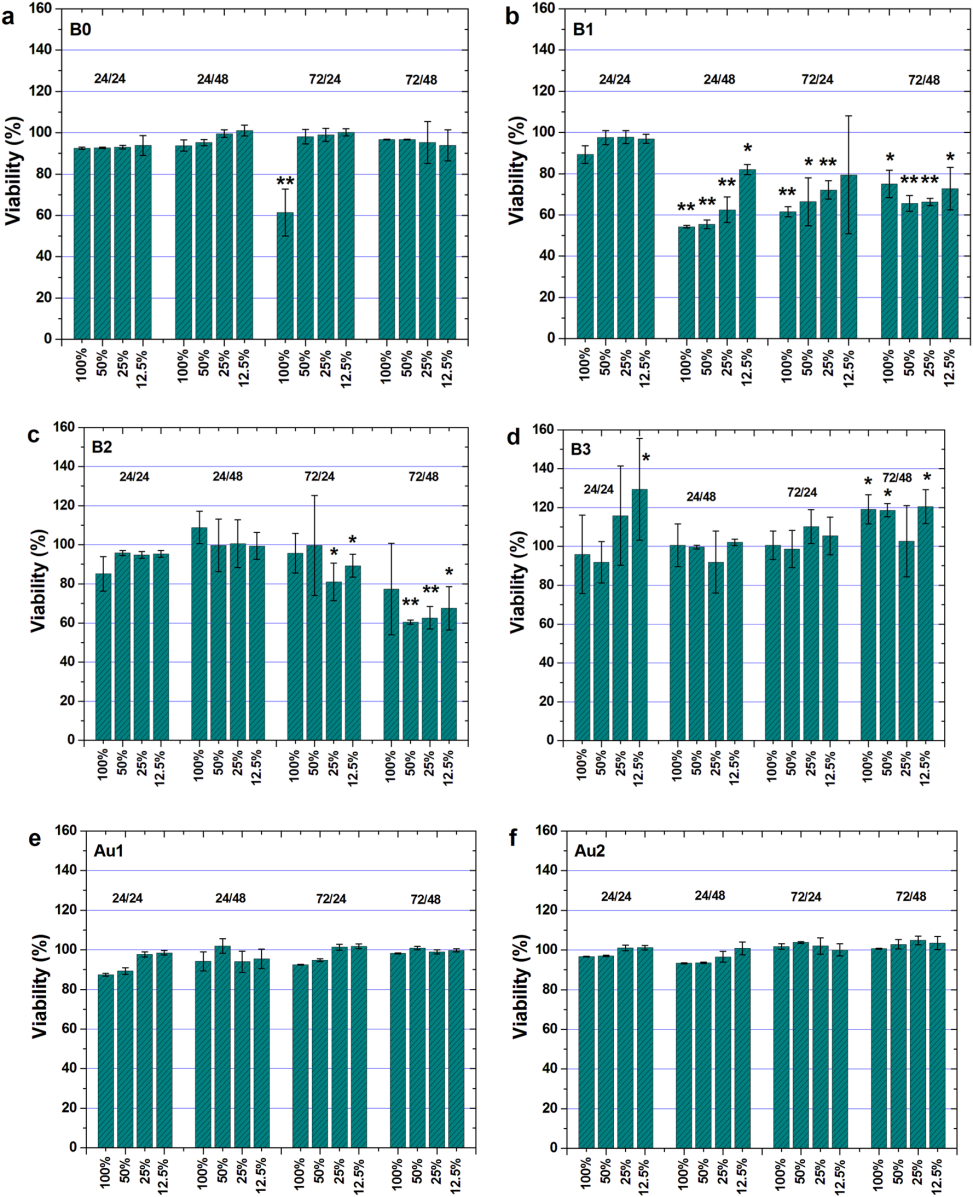
Cytotoxicity of U2-OS cells cultured in extraction mediums after 24 and 48 h of incubation for alloys: (**a**) Ca_32_Mg_12_Zn_38_Yb_18_; (**b**) Ca_32_Mg_12_Zn_38_Yb_17_B_1_; (**c**) Ca_32_Mg_12_Zn_38_Yb_16_B_2_; (**d**) Ca_32_Mg_12_Zn_38_Yb_15_B_3_; (**e**) Ca_32_Mg_12_Zn_38_Yb_16_B_1_Au; (**f**) Ca_32_Mg_12_Zn_38_Yb_14_B_2_Au_2_. Data are the mean ± SD of three independent experiments. *p < 0.05 and **p < 0.01 versus control.

The biocompatibility of the CaMgZnYbBAu alloys was studied using the MTT assay, which is an indicator of cell viability, proliferation, and cytotoxicity (see Fig. 5). It is known that osteoblastic cell adhesion, their growth, and proliferation were correlated with surface roughness. Moreover, the interactions between osteoblasts and orthopedic biomaterials depend on the development of bone-implant interfaces. Because of that, osteoblastic adhesion is very important for the first bone-biomaterial interaction^45,46^.

The colorimetric MTT assay is characterized by high sensitivity and therefore it can be used to study a large number of different samples^21^. The amount of MTT assay is proportional to the number of metabolically active cell cultures. It should be mentioned that cells have limited ability to proliferate. They can divide about 50 times with a culture medium that is frequently changed. Samples with absorption values less than 50% of division activity are considered toxic^21^. In our studies, the biocompatible effect of the samples was studied by indirect contact^19^. The extracts of the samples (in a form of plates) after 24 and 72 h were added to the cells. The time for cell incubation was 24 and 48 h. In addition, the content of the extracts was 100, 50, 25 and 12.5%, adequately. The results of the cytotoxicity tests showed that the Ca_32_Mg_12_Zn_38_Yb_15_B_3_ alloy was characterized by the highest cell viability (Fig. 5d). Cell viability greater than 100% was visible both for 24 and 48 h of incubation and for 24 and 74 h of extraction. Slightly lower cell viability was observed for the Ca-based alloy with 2 at.% Au (Fig. 5f). The cytotoxicity results were in agreement with the corrosion resistance of the Ca_32_Mg_12_Zn_38_Yb_15_B_3_ and Ca_32_Mg_12_Zn_38_Yb_14_B_2_Au_2_ alloys. It can also be stated that the alloy without boron is toxic and cannot be used as a potential biomaterial. Hakki et al.^10^ studied boron effect of the addition on the cell proliferation. The impact of B concentrations on cell viability was evaluated at 24 and 48 h using an MTT assay. The authors stated that boron plays an important role in bone metabolism^11^. The effect of Au was commented on in^47^ in which its influence on Fe-based alloy properties was investigated. The authors^47^ assessed that the addition of 5 wt.% Au in Fe-based alloys is an alternative to bioresorbable materials due to mechanical properties and biocompatibility understood, among others, as a cell vialibity and with the appropriate degradation rate.

Approximately a hundred data are related to the biocompatibility of bioresorbable Mg-based alloys^48–50^. Article^50^ presents data on mitochondrial metabolic activity of cells exposed, among others, to pure Mg alloy for 24 h. Analysis using MTT and LIVE/DEAD assays indicated metabolic acticity over 80% that was summerized as high cell viability^50^. In another work^51^, cytotoxicity studies showed that Mg–1.2Ca–1Zn alloy extraction medium resulted in higher cell viability than composition with Bi addition. The authors^51^ referred to the study^52^, in which the in vitro cytotoxicity of Y, Nd, Dy, Pr, Gd, La, Ce, Eu, Li, and Zr was analyzed. According to work^52^, the cytotoxicity of Ce and La is strongly associated with ionic radii, therefore, the biocompatibility of alloys with the addition of Bi was lower compared to the Mg-1.2Ca-1Zn alloy^51^. In this work, the cytotoxicity of Ca-based alloys could also be related with the differences between the ionic radii of B and Au. In the work^53^ there are also results of cytotoxicity studies on bioresorbable Zn-Mg alloys. According to the plot of cell viability measured through the MTT test presented in^53^, the viability of cells after 24 h in the Zn–1Mg alloy was slightly below 100%. After 72 h, the value increased above 100%^53^. Moreover, in paper^49^ the Ca-P coatings on the Mg-Zn-Zr alloy were investigated. According to cytotoxicity studies, after three days of incubation, the Ca-P coating had a favorable effect on biocompatibility compared to the uncoated alloy^49^.

**Figure 6:**
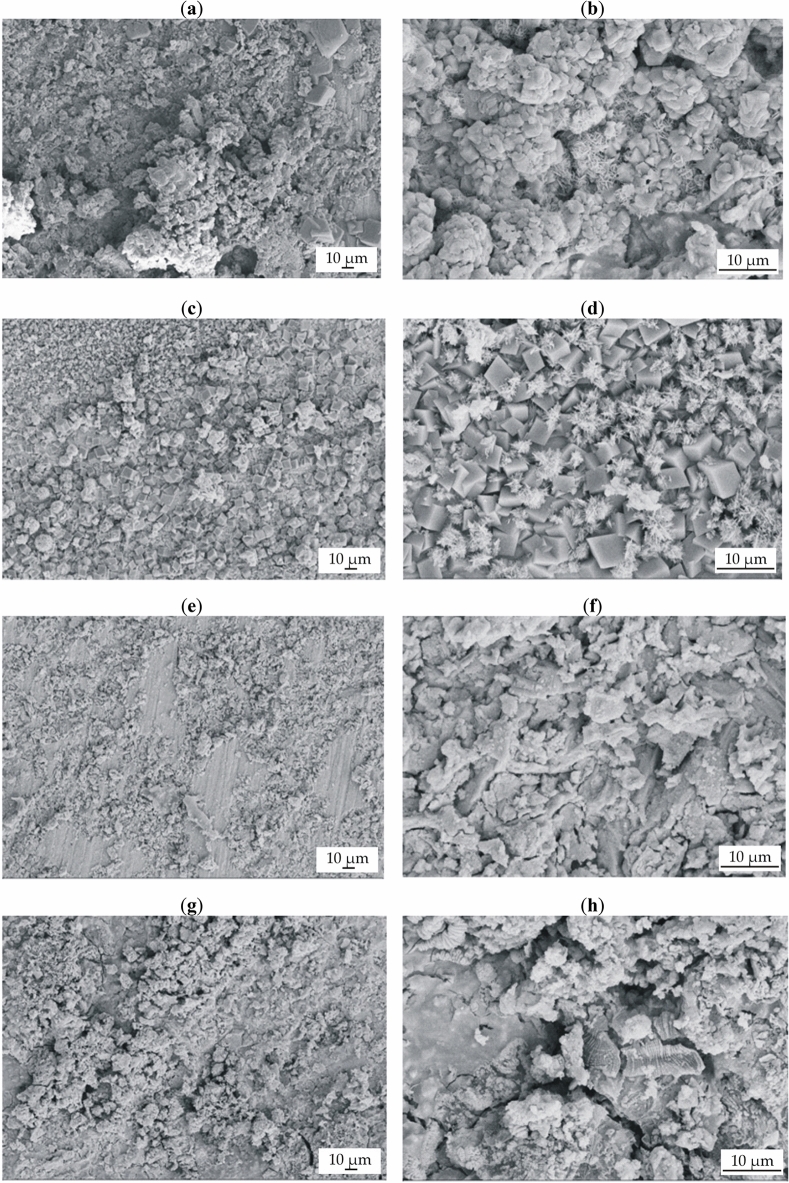
SEM images of samples’ surfaces with corrosion products after 48 h of immersion in Ringer’s solution at 37 °C for: (**a,b**) Ca_32_Mg_12_Zn_38_Yb_15_B_3_; (**c,d**) Ca_32_Mg_12_Zn_38_Yb_14_B_2_Au_2_, and after immersion in PWE fluid: (**e,f**) Ca_32_Mg_12_Zn_38_Yb_15_B_3_; (**g,h**) Ca_32_Mg_12_Zn_38_Yb_14_B_2_Au_2_.

After 48 h of immersion in Ringer’s solution and PWE fluid at 37 °C, the surface morphologies of the samples with corrosion products were microscopically observed. The analysis aimed to examine alloys with a lower corrosion rate and a higher cell viability, such as Ca_32_Mg_12_Zn_38_Yb_15_B_3_ and Ca_32_Mg_12_Zn_38_Yb_14_B_2_Au_2_. The SEM images are presented in Fig. 6. Microcracks were observed in all alloy samples, due to dehydration during the drying of the samples. In addition, the surfaces of the alloys were covered with corrosion products, and some corrosion products were also observed to fall off of the surface. It can also be seen that the entire surface of the samples with 3 at.% of boron and with 2 at.% of Au after immersion in PWE was not covered with corrosion products (Fig. 6e,g). Furthermore, the shapes of the corrosion products were different depending on the type of corrosive solution (Ringer’s or PWE solution). The samples exposed to Ringer’s solution were covered with cuboid-shaped and needle-shaped corrosion products compared to the samples exposed to PWE fluid, where flaky-shaped corrosion products can be seen.

**Figure 7:**
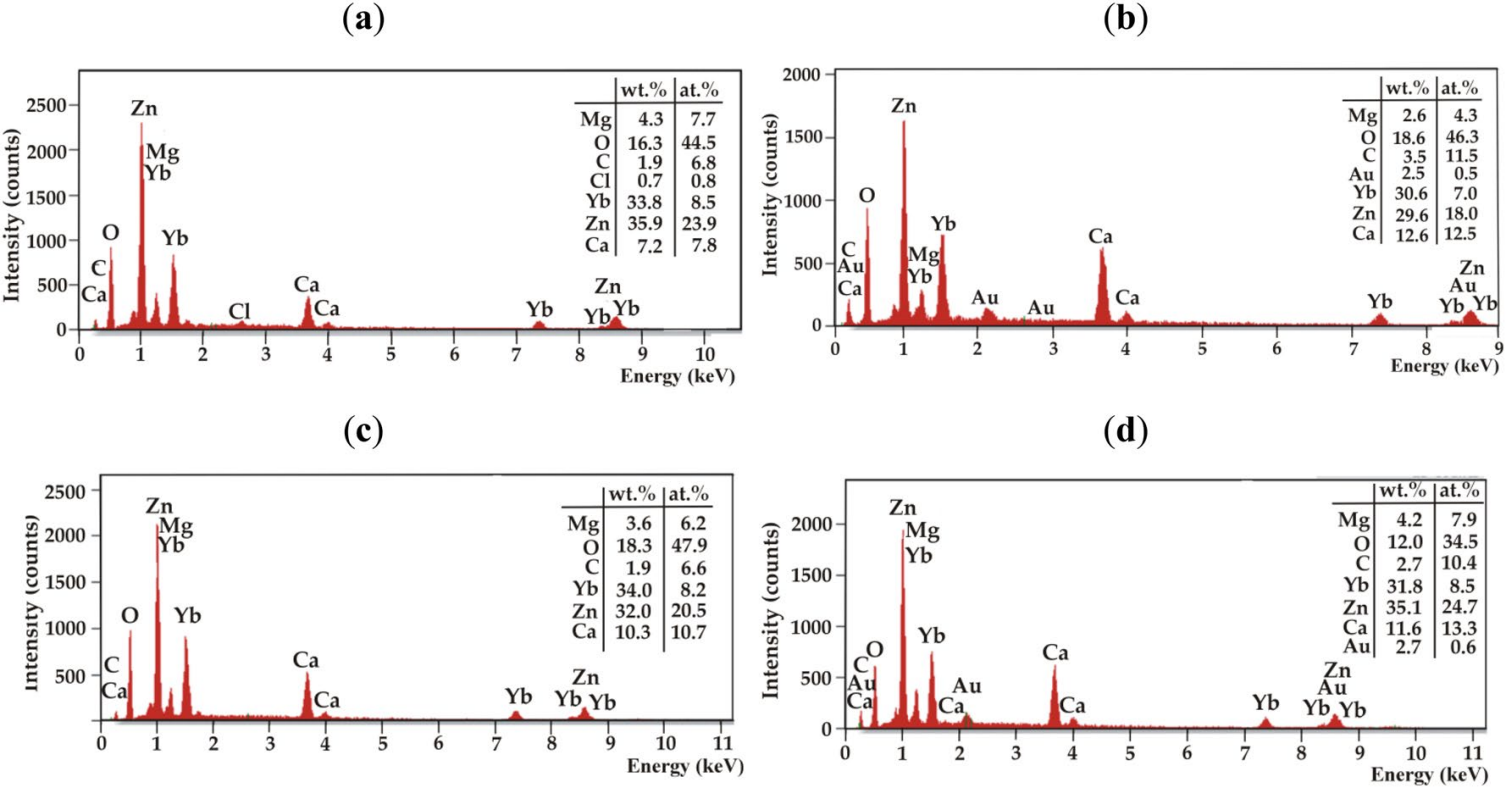
EDS analysis for corrosion products after 48 h of immersion in Ringer’s solution at 37 °C for: (**a**) Ca_32_Mg_12_Zn_38_Yb_15_B_3_; (**b**) Ca_32_Mg_12_Zn_38_Yb_14_B_2_Au_2_, and after immersion in PWE fluid for: (**c**) Ca_32_Mg_12_Zn_38_Yb_15_B_3_; (**d**) Ca_32_Mg_12_Zn_38_Yb_14_B_2_Au_2_.

**Figure 8:**
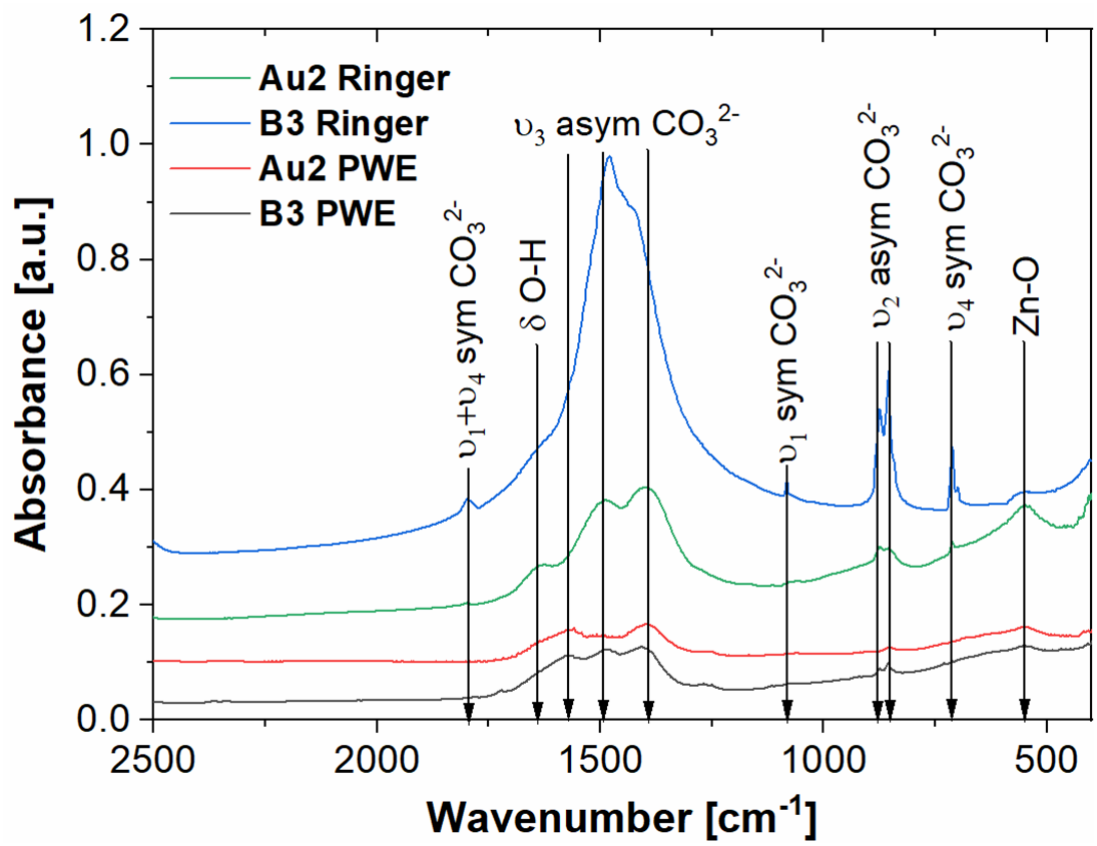
FTIR spectra of Ca_32_Mg_12_Zn_38_Yb_15_B_3_ and Ca_32_Mg_12_Zn_38_Yb_14_B_2_Au_2_ samples after immersion test in Ringer’s and PWE solutions with marked characteristic vibrations.

In addition, EDS analysis and FTIR tests were performed for the corrosion products of the alloys and solutions mentioned above (Figs. 7 and 8). The results of the EDS analysis showed that Zn, Yb, and O are the dominant constituent elements of the particles. There were also low-intensity peaks from Mg, Ca, C, and Cl. The lower intensity of chloride ions in corrosion products for both Ca-based alloys may suggest that these alloys were corrosion resistant.

The obtained FTIR spectra confirmed the presence of carbonates on the surface of the studied plates after 7 days of the immersion test in both PWE and Ringer’s solutions. The peak observed at 1645 cm^−1^ for the B3 sample (Ca_32_Mg_12_Zn_38_Yb_15_B_3_) kept in Ringer’s solution is associated with the δ O–H vibration of water^54^, while the highly visible broad peak around 854 cm^−1^ (from the Zn–O vibration) confirmed, that the corrosion products presented on the surface are probably related to the ZnCO_3_^55,56^. The characteristic of carbonates υ_1_ symmetric, υ_2_ asymmetric and υ_4_ symmetric vibrations were observed at 1082, 874, 854 and 713 cm^−1^^54^. Interestingly, the υ_3_ asymmetric vibrations of CO_3_^2−^ appeared at different wave numbers for analyzed samples. For example, sample B3 after immersion in the Ringer’s solution was characterized by very broad peaks at 1575, 1485 and 1405 cm^−1^, while the Ca_32_Mg_12_Zn_38_Yb_14_B_2_Au_2_ sample immersed in the same solution showed the υ_3_ asymmetric vibrations observed at 1490 and 1402 cm^−1^. This phenomenon is related to differences in the complex crystal structure of the resulting corrosion products. The same findings were described years ago by Lee and Condrate^57^. The carbonate ion lies in a lower symmetry than that of unperturbed carbonate. Although only two bands can be observed for free carbonate ion, for the carbonates (such as zincum carbonate), large splitting can be observed. Accordingly, the structure and chemical composition of the formed carbonates are different for all samples and depend on both the chemical composition of the alloys and the solution.

## Conclusions

The purpose of the work was to study the biocompatible effect and corrosion behavior of the Ca_32_Mg_12_Zn_38_Yb_18−x_B_x_ (x = 0, 1, 2, 3 at.%) and Ca_32_Mg_12_Zn_38_Yb_18−2x_B_x_Au_x_ (x = 1, 2 at.%) alloys. The results of the investigations allowed us to draw the following conclusions:

- The Au addition of 1 and 2 at.% in CaMgZnYbB alloys and 3 at.% of B in CaMgZnYb improved corrosion resistance in PWE solution compared to Ca_32_Mg_12_Zn_38_Yb_18_ because of lower values of *j*_corr_ and higher *R*_p_. The B addition of 1 and 2% negatively affected the corrosion properties of the Ca_32_Mg_12_Zn_38_Yb_18_ alloy in PWE solution.
- The surface roughness analysis has shown that the lowest roughness values (*R*_a_ and *R*_s_ equal to 1.09 and 1.39 µm, respectively) were obtained for the alloy with 3 at.% of B. According to the results of the electrochemical measurements, the B addition of 3 at.% was more corrosion resistant compared to the addition of 2 and 1 at.% of B. The results of cytotoxicity tests using the MTT assay also showed that the alloy with 3 at.% of B was characterized by the highest cell viability, over 100% for both 24 and 48 h of incubation.
- The FTIR results indicated the presence mainly of carbonates with different structure and chemical composition (due to different alloy and solution compositions), on the surfaces of both the studied Ca_32_Mg_12_Zn_38_Yb_15_B_3_ and Ca_32_Mg_12_Zn_38_Yb_14_B_2_Au_2_ samples studied immersed in Ringer’s solution and PWE fluid.

## Data Availability

The data and material generated during and/or analyzed during the current study are available from the corresponding author on reasonable request.
